# Having versus not having social interactions in patients diagnosed with depression or social phobia and controls

**DOI:** 10.1371/journal.pone.0249765

**Published:** 2021-04-14

**Authors:** Jeanette Villanueva, Andrea H. Meyer, Thorsten Mikoteit, Jürgen Hoyer, Christian Imboden, Klaus Bader, Martin Hatzinger, Roselind Lieb, Andrew T. Gloster

**Affiliations:** 1 Department of Psychology, Division of Clinical Psychology and Intervention Science, University of Basel, Basel, Switzerland; 2 Department of Psychology, Division of Clinical Psychology and Epidemiology, University of Basel, Basel, Switzerland; 3 Psychiatric Hospital of the University of Basel, Center for Affective, Stress and Sleep Disorders, University of Basel, Basel, Switzerland; 4 Institute of Clinical Psychology and Psychotherapy, Dresden University of Technology, Dresden, Germany; 5 Psychiatric Services Solothurn and University of Basel, Basel, Switzerland; 6 Private Clinic Wyss, Muenchenbuchsee, Switzerland; 7 Psychiatric Hospital of the University of Basel, Center for Psychosomatics and Psychotherapy, University of Basel, Basel, Switzerland; University of Belgrade, SERBIA

## Abstract

Humans need meaningful social interactions, but little is known about the consequences of not having them. We examined meaningful social interactions and the lack thereof in patients diagnosed with major depressive disorder (MDD) or social phobia (SP) and compared them to a control group (CG). Using event-sampling methodology, we sampled participants’ everyday social behavior 6 times per day for 1 week in participants’ natural environment. We investigated the quality and the proportion of meaningful social interactions (when they had meaningful social interactions) and degree of wishing for and avoidance of meaningful social interactions (when they did *not* have meaningful social interactions). Groups differed on the quality and avoidance of meaningful social interactions: Participants with MDD and SP reported perceiving their meaningful social interactions as lower quality (in terms of subjective meaningfulness) than the CG, with SP patients reporting even lower quality than the MDD patients. Further, both MDD and SP patients reported avoiding meaningful social interactions significantly more often than the CG. Although the proportion of meaningful social interactions was similar in all groups, the subjective quality of meaningful social interactions was perceived to be lower in MDD and SP patients. Future research might further identify what variables influenced the reinforcement of the MDD and SP patients so that they engaged in the same number of meaningful social interactions even though the quality of their meaningful social interactions was lower. Increasing awareness of what happens when patients do or do not have meaningful social interactions will help elucidate a potentially exacerbating or maintaining factor of the disorders.

## Introduction

Social interactions played an essential role in the evolution of humans [1–6]. Due to the importance for humans to fulfil social needs [7], and the strong drive within humans to establish and preserve meaningful social relationships [8], a lack of meaningful social contact may result in negative sequelae. Therefore, both times of having meaningful social interactions and times of *not* having meaningful social interactions merit scientific attention. While social interactions might be experienced differently, instances of when people have no meaningful social interactions are much less explored, especially in clinical samples with difficulties in social interactions. Such difficulties might possibly contribute to less meaningful social interactions (e.g. through avoidance of eye contact in patients suffering from SP [9]), yet it is nevertheless possible to consider such interactions as meaningful, even if one “failed to perform”. Meaningful social interactions tend to be of higher quality [10] and are described as being subjective and having emotional, informational, or tangible impact, and to enhance one’s life [11].

Investigations of social interactions have found, for instance, that in patients with depression, the quantity of social interactions as retrospectively recalled did not differ from that of controls, but they reported their social interactions as being less close or suffering in quality [12–15]. Even when the quantity of social interactions was lower in participants with high depressive symptomatology, they also reported their social interactions as being less close [15–17]. Individuals with depression have been found to have lower social skills, reduced desire to communicate and cooperate, problems in understanding the thoughts or feelings of others, and deficits in performing social roles, possibly leading to stigma and social withdrawal [14, 18, 19]. In contrast, pleasantness has been shown to be associated with satisfying interpersonal experiences [20], and intimacy is associated with social closeness [21]. Therefore, the way social interactions are perceived, including how pleasant or intimate a social interaction is, might be impacted in patients suffering from depression, especially in social interactions that are considered meaningful. In other disorders, such as social phobia (SP), a marked and persistent fear of social or performance situations is, by definition, an integral part of the clinical picture [22, 23]. Individuals suffering from SP may have social skill deficits as well (e.g. difficulties starting or joining a social conversation or have increased expectations of failure in social contexts) [24], which may also impact how intimate or pleasant social interactions are experienced. While SP is associated with a reduced frequency of social interactions [25], it nevertheless remains to be seen what role pleasant and intimate social interactions play in patients suffering from SP.

While social interactions have been investigated in patients suffering from depression or social phobia, there is still a need to further explore instances of meaningful social interactions and when people have *no* meaningful social interactions. Thus, in this context, specific instances of when people have *no* meaningful social interactions are of interest, and less states of general loneliness or social isolation (e.g. [7]). While research on nonclinical samples has documented that community adults felt happier when they were with other people than when they were alone [26], how this plays out in *meaningful* social interactions and in clinical samples also remains to be seen.

Examining times of having meaningful social interactions and times of *not* having meaningful social interactions is especially important in disorders where strain on the social network is common [12, 15, 23, 27], and patterns of social withdrawal and difficulties in social interactions are characteristic [19, 28, 29], such as Major Depressive Disorder (MDD) or SP. Studying an affective and an anxiety disorder further allows for clinical specificity (i.e., whether findings are applicable specifically to one of these diagnoses or to all the groups, which could suggest relevance for affective and anxiety disorders).

Social interactions putatively involve uncomfortable aspects for patients with depression and SP. Given this aspect, it is unclear whether individuals with clinical diagnoses nevertheless wish for more meaningful social interactions when they do not experience them. Especially if social interactions turn into an aversive experience, patients may attempt to avoid them [30]. Due to the more negative and lower quality perception of their social interactions [12, 13], patients suffering from depression might not expect any close or high-quality social interactions. SP, on the other hand, is by definition characterized by a marked and persistent fear of social or performance situations, in which exposure to and scrutiny by others is possible. This leads to feared social situations being avoided or endured with intense anxiety or distress [22, 23]. Furthermore, the social behavior in individuals with either depression or SP has an effect on others, which often leads to these individuals themselves being avoided as interaction partners [31]. How these relationships can be observed when people have meaningful social interactions versus when they do not, however, is yet to be seen.

Understanding participants’ social behavior requires data collection in participants’ natural environment [32]. Implementing Event-Sampling-Methodology (ESM) allows the examination of participants’ natural social interaction choices and motivations, both when people have social interactions and when they do not. Thus, ecologically valid and more accurate data can be collected while capturing dynamic changes of variables [33]. Additionally, ESM is suitable and useful for the assessment of moods, thoughts, symptoms, and behaviors which change over time [32, 34, 35]. Since human memory is subject to recall bias [36] ESM also reduces the effect of recall bias through real-time data collection.

### Hypotheses

We investigated how *having* meaningful social interactions versus *not having* meaningful social interactions was perceived by patients with a diagnosis of MDD or SP, in comparison to a control group (CG). Overall, when participants *had* meaningful social interactions our hypotheses focused on 2 outcome measures: (1) Proportion of meaningful social interactions, whereby we hypothesized a smaller proportion of meaningful social interactions for participants suffering from MDD or SP, in comparison to the CG and (2) quality of meaningful social interactions, whereby we hypothesized that the quality would be lower in the MDD group, and in the SP group, comparing each group separately to the CG. When participants did *not* have any meaningful social interactions, our hypotheses focused on 2 other outcome measures: (3) wishing for meaningful social interactions, whereby we hypothesized that the MDD group and the SP group would report a higher level of wishing for a meaningful social interaction in comparison to the CG and (4) avoidance of meaningful social interactions, whereby we hypothesized that the MDD group and the SP group would report a higher level of avoidance of meaningful social interactions in comparison to the CG.

## Materials and methods

### Participants

Individuals diagnosed with a mental disorder (MDD, *N* = 118; or SP, *N* = 47) and individuals without a diagnosis of MDD or SP (CG, *N* = 119) were investigated. Participants were recruited through local advertising and treatment centers (university clinics and cooperating local practitioners) for clinical participants and via local advertising for the CG. Recruitment was done in Switzerland and Germany. All participants completed written informed consent procedures. Participants in the study were treated in accordance with international ethical standards and as approved by the Ethikkommission beider Basel, EKBB (Reference number for this study: Ethikkommission beider Basel, EKBB– 236/12). The groups were matched for age and sex. The mean age was 31.75 years (*SD* = 11.52, range: 18 to 63 years), and the majority of the participants were female (*n* = 66.5%). Consistent with the demographics of this region, the entire sample was of Western European descent. Further demographic information can be viewed in Table 1. For further information please see [37]. Inclusion criterion was age (18–65 years). Exclusion criteria were acute suicidality, current substance dependence, inability to understand German, and physical disabilities preventing participation (e.g., inability to see text on a smartphone or to hear the smartphone’s signal). Power calculations were done with the software G*Power. Power calculations were based on hypotheses specific for a larger study (for more information, please see [37]), believed to have the lowest effect size, i.e. on between-group comparisons which involve the SP group. The number of SP patients that could be feasibly recruited within the study period was amounted to 48 patients, and was thus a predefined constraint. Assuming a dropout rate of 5%, the expected number was 45 patients. Power analyses were based on this number and assumed alpha = .05, and resulted in power = .8, and a two-sided test. Based on a t-test for independent samples, a medium effect size (d = 0.5), and 45 SP patients, the sample size necessary to achieve.8 power was 111 subjects in each of the other groups (MDD and CG). For more information, please see [37].

**Table 1:** Demographic information (%) and sample characteristics [mean and standard deviation (SD)].

	MDD N = 118	SP N = 47	CG N = 119
Age in years			
Mean	32.7	28.3	32.2
Median	29.0	26.0	28.0
SD	12.0	7.8	12.0
Sex			
Male	33.9	34.0	32.8
Female	66.1	66.0	67.2
Years of education			
8–10	21.1	9.3	12.0
11–13	51.4	67.4	53.0
14+	27.5	23.3	35.0
Living arrangement			
Alone	22.9	21.3	30.3
Family/partner	60.2	55.3	49.6
Other	16.9	23.4	20.2
Employment Status			
Employed	52.5	38.3	57.1
Unemployed	46.6	61.7	39.5
Number of diagnoses			
0	0.0	0.0	90.8
1	45.8	44.7	6.7
2	29.7	27.7	1.7
3+	24.6	27.7	0.8
In therapy			
No	41.5	53.2	85.7
Yes	58.5	46.8	14.3

MDD = Major Depressive Disorder, SP = Social Phobia, CG = Control Group. Adapted from [37].

### Design and procedure

A 7-day event-sampling phase within a quasi-experimental, intensive, longitudinal study was studied. Participants carried a study-issued smartphone during this phase. Measures included in this paper were a subset of those used in the larger study. For further details on the procedure, please see [37].

### Assessment

All participants completed the Structured Clinical Interview for *DSM-IV* Axis I Disorders (SCID; [38]. We used the SCID-I (current diagnosis), which has moderate to excellent values for reliability and validity [39–41]. Diagnoses were rated on the Anxiety Disorders Interview Schedule severity rating scale [42]. The primary diagnosis (i.e., the diagnosis with the highest severity score) determined group assignment. ESM data were collected six times a day using signal-contingent ESM every 3 h (e.g., 8:00 a.m., 11:00 a.m., 2:00 p.m., 5:00 p.m., 8:00 p.m., and 11:00 p.m.). We also discussed concepts such as meaningfulness with participants before they started into the ESM data collection week. Consistent with the subjective nature of this concept [11], we emphasized that it was up to them to decide what was meaningful, and that a predetermined length or interaction partner was not necessary. To further clarify this to the participants and to gather more information on what they perceived to be meaningful, each participant articulated several situations they considered to be meaningful social interactions. After it was confirmed that there were no questions left, participants entered the ESM phase. During this week participants were asked to report on the occurrence or nonoccurrence of meaningful social interactions since the last reminder. When participants had any social interactions, items followed about (a) proportion: “How many social interactions were meaningful to you?,” dichotomized into *none* versus *one or more* and (b) social interaction quality: “Did you perceive the interaction as pleasant?,” on a scale of 0–100 (*unpleasant* to *pleasant*), and “How would you estimate the level of intimacy of the interaction?,” on a scale of 0–100 (*not intimate* to *intimate*). Based on earlier research ratings of pleasantness and intimacy were combined into a rating representing social interaction quality [20, 21]. When participants did *not* have any meaningful social interactions, items followed about (a) wishing for a meaningful social interaction: “Did you wish for such a [meaningful] social interaction?” (No, Yes), and (b) avoiding a social interaction: “Did you avoid such a [meaningful] social interaction?” (No, Yes). Items were all chosen a priori and adapted to the ESM format used in this study (by adding an indication of the time frame since the last reminder: “Since the last beep, […]”). Items stemmed from previous ESM studies [16, 43, 44] and were based on a functional analysis of social interactions [45] due to their individual nature.

### Statistical analysis

Data were analyzed using R 1.2.1335 [46]. The following packages were used for the data-analysis: car [47], lme4 [48], effects [49], predictmeans [50], boot [51, 52], ICCbin [53]. Data were included in the analyses if a participant answered at least 50% of the smartphone reminders. Six participants completed less than 50% of ESM time points and were therefore removed from the data set. Those six participants did not differ from the rest of the sample in terms of age, group assignment, or sex. All outcome variables varied within participants across time points whereas the predictor (diagnostic group) varied between participants.

In consideration of the study design and the structure of the data, the data was analyzed using multilevel models. Multilevel models consider the variability of ESM based measures within subjects, unequal group sizes, and missing data. These models are therefore well suited to analyze data collected from ESM studies, which are repeated measures with interdependent observations of data nested within individuals. Generalized linear mixed models (GLMMs) were therefore implemented for binary outcomes (Hypotheses 1, 3, and 4), and linear mixed models (LMMs) were implemented for continuous outcomes (Hypothesis 2). For all four hypotheses, the GLMM contained as sole fixed effect the diagnostic group which was assessed at the upper level of the participants, plus a random intercept to account for the dependency among repeated measures within subjects. Calculations were based on a sample of 5,609 (MDD: *n* = 2222; SP: *n* = 861; CG: *n* = 2526) instances when participants had meaningful social interactions, and a sample of 1,356 (MDD: *n* = 550; SP: *n* = 221; CG: *n* = 585) instances when participants had no meaningful social interactions. The CG was the reference group for models comparing the diagnostic groups (MDD and SP) to the CG. When comparing the diagnostic groups to each other, MDD was the reference group. GLMMs contained a random intercept to account for the dependency among repeated measures within subjects. Considering the nested structure of the data set, outcomes were state measures in all models. Each data point of each participant was put in relation to the group (i.e. six scores per day per person). Thus, by avoiding to collapse scores into aggregate variables, using multilevel models allowed for fine-grained analyses.

## Results

**Fig 1:**
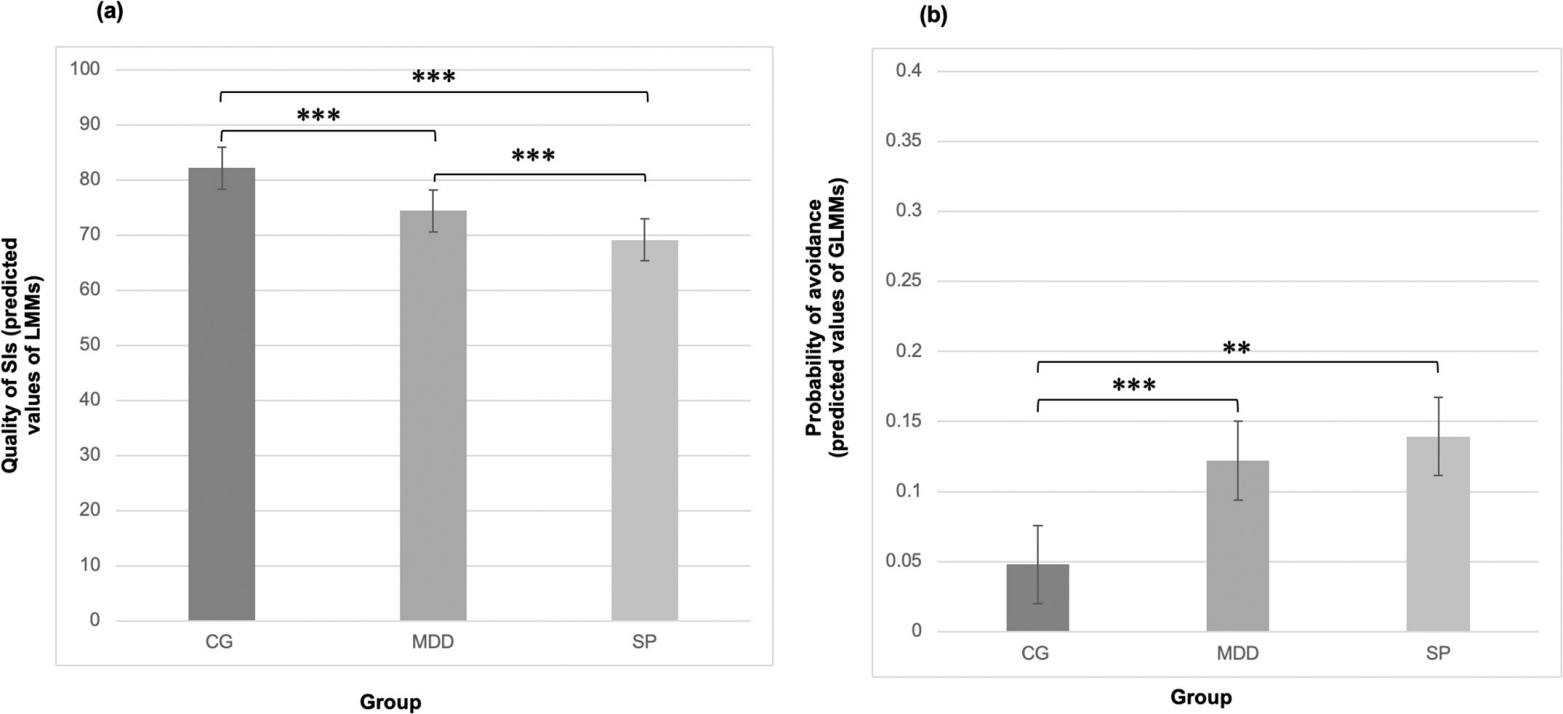
Results of the linear mixed models (LMMs) and the generalized linear mixed models (GLMMs). Differences in (a) quality of social interactions, on a scale of 0–100, and (b) avoidance of social interactions (dichotomous, 0 or 1), depending on group (major depressive disorder [MDD] diagnosis, social phobia [SP] diagnosis, control [CG]). **p < .01. ***p < .001.

We compared the CG to each of the diagnostic groups (CG vs. MDD, CG vs. SP) and then compared the two diagnostic groups to each other (SP vs. MDD) for all analyses. Overall, participants responded to 92.3% of queried assessments. Average severity of patients suffering from MDD (5.53) did not differ significantly from average severity of patients suffering from SP (5.43). For demographic information and sample characteristics see Table 1, as adapted from [37]. For the summarized results for Hypotheses 1 to 4 see Table 2 and Fig 1.

**Table 2:** Differences in the proportion and quality of experienced social interactions, and differences in the level of wishing for a social interaction and avoidance of social interactions.

Social interaction experience	Outcome	MDD vs. CG		SP vs. CG		SP vs. MDD	
		OR (95% CI) / β (*SE*)	*p*	OR (95% CI) / β (*SE*)	*p*	OR (95% CI) / β (*SE*)	*p*
Social interactions experienced	Proportion of meaningful social interactions	0.88 (0.61, 1.26)	.47	0.96 (0.59, 1.55)	.85	1.09 (0.68, 1.78)	.73
	Quality of social interactions	-7.74 (1.45)	< .00***	-13.01 (1.92)	< .00***	-5.26 (1.93)	< .00***
No social interactions experienced	Wishing for a social interaction	0.99 (0.43, 2.28)	.98	1.97 (0.64, 6.09)	.27	1.99 (0.68, 1.77)	.23
	Avoidance of social interactions	3.36 (1.44, 8.65)	.006**	4.16 (1.30, 14.53)	.02*	1.23 (0.38, 3.95)	.71

OR: Odds ratio; CI: confidence interval; β: Beta coefficient; SE: Standard error; MDD: major depressive disorder; SP: social phobia; CG: control group. ORs and CIs refer to generalized linear mixed model results; β and SEs refer to linear mixed model results. *p*-values in bold writing indicate significant results.

**p < .01.

***p < .001.

All outcome variables belong to the intraindividual level of the analysis. Note that Intraclass Correlation (ICC) for the outcomes was extremely small (for Hypotheses 1, 3, and 4 < .001; for Hypothesis 2 = 0.28) and thus a generalized linear model (GLM) would have led to identical results. For Hypotheses 1, 3, and 4 ICCs were calculated according to the aov method for binary outcomes [52, 53].

Hypothesis 1 presumed a smaller proportion of meaningful social interactions for participants suffering from MDD or SP, in comparison to the CG. The overall proportion of meaningful social interactions was rather high in all three groups (CG: 81.2%; MDD: 80.2%; SP: 79.6%) and all groups reported only rarely having had more than one meaningful social interaction per 3-hour time window (4.71% for the MDD group, 5.82% for the SP group, and 6.89% for the CG) For more information about the absolute and relative numbers, please see S1 Table. GLMM results indicated that the ratio of proportions did not differ between the groups (MDD vs. CG: OR = 0.88, *p* = .45, 95% CI, [0.61, 1.25], slope: -0.06; SP vs. CG: OR = 0.96, *p* = .84, 95% CI [0.59, 1.55], slope: -0.10; SP vs. MDD: OR = 1.09, *p* = .73, 95% CI [0.68, 1.78], slope: 0.09).

Hypothesis 2 investigated whether the quality of meaningful social interactions would be lower in the two diagnostic groups (MDD and SP) compared to the CG. Results suggest that both MDD and SP participants perceived their meaningful social interactions to be significantly lower in quality compared to the CG. MDD patients report the quality of their meaningful social interactions to be lower by 7.74, compared to the CG, (MDD vs. CG: β = -7.74, *SE* = 1.45, *p* < .00, slope: -6.17), while SP patients report the quality of their meaningful social interactions to be lower by 13.01, compared to the CG (SP vs. CG: β = -13.01, *SE* = 1.92, *p* < .00, slope: -8.87). Participants suffering from SP reported even lower quality of meaningful social interactions than participants suffering from MDD, this by 5.26 (SP vs. MDD: β = -5.26, *SE* = 1.93, *p* < .00, slope: -5.26). Further, we have recalculated this analysis with pleasantness and intimacy separately. The result remains the same (intimacy: MDD vs. CG: β = -4.36, SE = 1.59, p = .006; SP vs. CG: β = -10.10, SE = 2.11, p < .00); pleasantness: MDD vs. CG: β = -11.12, SE = 1.51, p < .00; SP vs. CG: β = -16.00, SE = 2.01, p < .00). For more information about the means and standard deviations of both pleasantness and intimacy, please see S2 Table.

Hypotheses 3 and 4 concerned instances when participants reported *not* having had any meaningful social interactions. Hypothesis 3 presumed that the patients (MDD and SP) would report a higher level of wishing for a meaningful social interaction than the CG. Overall, wishing for a meaningful social interaction was comparable across groups (MDD vs. CG: OR = 1.23, *p* = .48, 95% CI [0.68, 2.28], slope: 0.21; SP vs. CG: OR = 1.75, *p* = .16, 95% CI [0.81, 3.85], slope: 0.56; SP vs. MDD: OR = 1.42, *p* = .37, 95% CI [0.65, 3.05], slope: 0.69). For more information about the absolute and relative numbers, please see S3 Table.

Hypothesis 4 investigated whether the participants suffering from MDD or SP would report a higher level of avoidance of meaningful social interactions in comparison to the CG. Results suggest that participants suffering from MDD or SP avoided meaningful social interactions significantly more often compared to the CG. MDD patients were 2.96 times more likely to avoid meaningful social interactions compared to the CG (MDD vs. CG: OR = 2.96, *p* = .001, 95% CI [1.54, 5.86], slope: 0.43), while SP patients were 3.66 times more likely to do so (SP vs. CG: OR = 3.66, *p* = .003, 95% CI [1.57, 8.74], slope: 0.55). Interestingly, there were no differences between the MDD and SP groups (SP vs. MDD: OR = 1.16, p = .73, 95% CI [0.49, 2.76], slope: 0.21). For more information about the absolute and relative numbers, please see S4 Table.

## Discussion

This study examined participants diagnosed with MDD or SP and a CG during an intensive 1-week longitudinal investigation in their natural environment. Through using ESM to investigate two highly prevalent and relevant clinical groups (MDD and SP) and comparing them to a CG, we aimed to surmount the limitations of cross-sectional testing in an ecologically valid manner, while simultaneously testing for clinical specificity. Results suggest three main findings: First, when participants *had* meaningful social interactions, the diagnostic groups (MDD and SP) reported a lower quality of meaningful social interactions than controls, with participants suffering from SP reporting even lower quality than participants suffering from MDD. Additionally, the proportion of meaningful social interactions was comparable among the different groups. When participants did *not have* any meaningful social interactions, patients (MDD and SP) reported a significantly higher level of avoiding meaningful social interactions than the CG, with the two diagnostic groups not differing from each other. Neither the proportion of meaningful social interactions nor the level of wishing for a meaningful social interaction differed between the MDD group, the SP group, and the CG.

### Having meaningful social interactions: Proportion and quality of meaningful social interactions in patients diagnosed with MDD or SP and controls

Given that social interactions are associated with higher negative affect and lower positive affect in patients diagnosed with MDD and SP [12, 54–56], one might expect participants with MDD and SP to show a different proportion of meaningful social interactions compared to a CG. However, our result showed no difference in the proportion of meaningful social interactions between the groups. This is consistent with ESM research showing no differences in the proportion of social interactions in participants with depression compared to controls [12, 13, 55] but is in contrast to earlier research showing reduced proportions of social interactions in participants with SP compared to controls [25]. Discrepant results regarding the proportion of social interactions might be due to methodology (retrospective self-report during an interview [25] vs. ESM in the present study). Further, the present study specifically queried for meaningful interactions, whereas previous studies either did not differentiate or did not report on whether the social interactions they investigated were meaningful. Similarly, meaningful social interactions may or may not have elicited feelings of scrutiny in participants. This is especially important with patients diagnosed with SP: Despite part of the definition of SP [22, 23] possibly leading to an expectation of a lower proportion of meaningful social interactions in this group, the definition of SP includes situations that involve scrutiny. Thus, patients suffering from SP do not always have phobic social interactions. The current study therefore provides new insights into SP beyond phobic social interactions.

Despite the absence of a difference in the proportion of meaningful social interactions between the groups, participants suffering from MDD or SP reported significantly lower quality of meaningful social interactions compared to the CG. This is consistent with previous ESM [12, 13, 16] and non-ESM research [14, 15, 17] showing that patients with depression perceive social interactions as being less close or suffering in quality. Again, previous studies not differentiating or reporting on whether the social interactions they investigated were meaningful complicates comparisons with other studies. Multiple processes could contribute to perceiving meaningful social interactions as “lower quality”, including patients with depression showing negative reactivity specifically after social interactions (i.e., patients display increased negative affect after social interactions; [57], a tendency of a negatively biased perception of themselves and others [58, 59], or emotion suppression to downregulate negative emotions [60–63].

Interestingly, participants suffering from SP reported even lower quality of meaningful social interactions compared to participants suffering from MDD. There might be multiple reasons for this: First, individuals with SP fear specific social situations greatly [22, 23] and since fear is rarely experienced as positive, fear might impact the quality of their social interactions. Second, individuals with SP possibly engage more in constant monitoring of threat and anxiety during social interactions, which can disrupt recognition and acknowledgment of rewards during this time [64]. Constant monitoring might arise due to a tendency to engage more in negative self-referent and self-evaluative thoughts [65–67], perceiving the interaction partner as more dominant [59], interpreting ambiguous social events in a negative way and mildly negative events in a catastrophic fashion [68], ruminating about possible social failures and possible devaluation by others after social interactions [69, 70], which maintain distress and negative self-appraisals [26], or seeing social outcomes as information about expectations that others might have, rather than information about one’s own competence [71]. Additionally, the reported social interactions might or might not include performance situations, since we enquired about meaningful social interactions. This is an important point, since fear of situations of possible scrutiny and fear of more general social interactions are different aspects of the disorder [72].

### Not having meaningful social interactions: Wishing for and avoiding meaningful social interactions in patients diagnosed with MDD or SP and controls

Our results showed that neither participants suffering from MDD nor participants suffering from SP wished for meaningful social interactions any differently from the CG. On the one hand, this is in contrast to earlier research, indicating that deficits in social functioning (as they are existent in patients with MDD or SP) may decrease the potential to engage in social interactions [18]. On the other hand, it has been shown in past research that patients perceive their social interactions to be more negative [12, 13]. In the light of this result, patients might not wish for more social interactions because they believe there is no reason to expect any high-quality social interactions, or possibly their social interactions were fulfilling and meaningful as they were. Future research should test both advantageous and dysfunctional antecedents and consequences of being alone to investigate this finding further.

Participants suffering from MDD or SP avoided meaningful social interactions more often than the CG. Aversive thoughts may evoke behavior to avoid and escape the thought (e.g., avoidance of romantic relationships after a breakup to avoid anxiety and self-referential thoughts; [30]). It is therefore possible that participants suffering from MDD avoided meaningful social interactions to avoid social-interaction-related thoughts, thus possibly not experiencing the reinforcing aspects of social interactions [30]. Participants with SP may have avoided meaningful social interactions because the quality of their social interactions was low or because of limited interpersonal skills, due to habitual avoidance.

It is also possible that, because they perceived their social interactions as lower quality, each individual social interaction was thus less reinforcing for the diagnostic groups, resulting in a vicious cycle [30, 59]. Thus, since there was a comparable proportion for participants suffering from MDD or SP, and the CG, but the participants suffering from MDD or SP reported a lower quality of social interactions, it cannot be the quality of social interactions alone that had a reinforcing impact on their social interactions [73], otherwise the proportion of social interactions would have been lower for participants suffering from MDD or SP than for the CG [73]. According to our operationalization of quality of social interactions, interactions that feel more pleasant and more intimate might impact the perceived quality of social interactions. Both clinical work and future research might attempt to identify further variables that contribute to the different relative rates of reinforcement.

### Limitations

This study has five main limitations: First, although ESM is the gold standard for capturing real-life behavior in context in part because participants can report their behaviors and feelings more accurately than with questionnaires [74], in this case it was still a self-report of self-selected social interactions. Second, participants decided themselves what a meaningful social interaction was, which might have differed across participants. It is difficult to imagine how this component could be standardized, but it is nevertheless important to keep in mind that it was subjective meaningfulness that was assessed. Third, these meaningful social interactions were not defined in terms of valence, and thus a meaningful social interaction could have been associated with negative or positive emotions for a participant. This is especially important in regard to the negative emotional bias of people diagnosed with MDD. Future research might consider varying assessment density by increasing the assessment frequency or including nonmeaningful social interactions to explore whether our findings persist in all social situations. Fourth, the ESM data collection period lasted 7 days. While this period is shorter than some other ESM studies (e.g. [35]), we aimed to keep time and resource burden to a minimum, especially on the side of the patient. Further, we are confident that 7 days suffice to capture occurrences and non-occurrences of often frequently happening events (such as social interactions) and the corresponding thoughts and feelings. Since we captured participants’ waking time during every day of the week, there was no reason to believe that extending the data collection period would yield differing results. Fifth, the distribution of the participants to the groups analyzed in this study (i.e., MDD, SP, CG) was based on the primary diagnosis.

## Conclusions

The present study provides new insights into meaningful social interactions of individuals diagnosed with MDD and SP, and also into the experience of not having meaningful social interactions. Participants were asked about their experiences when *having* a meaningful social interaction (proportion and quality of social interactions) and when they did *not have* any meaningful social interactions (wishing for and avoidance of social interactions), providing over 10,000 data points and thus enabling us to examine a reliable part of everyday life that cannot be tested by other means. On the one hand, this study has practical implications. A stronger focus on what reinforces or encourages patients to engage in meaningful social interactions, as opposed to avoiding them, might putatively lead to a richer and more fulfilling life. This might possibly happen because meaningful social interactions tend to have emotional, informational, or tangible impact on one’s life [11]. On the other hand, it also contributes to theoretical knowledge: Investigating how meaningful social interactions and a lack thereof are perceived is an important step in discovering how social interactions contribute to mechanisms that maintain or alleviate MDD and SP.

Social interactions are not always easy. However, if people approach them in such a way that they support rather than burden, they might prove to be helpful in times of need. Contrary to common belief, symptoms do not necessarily need to go away before one can engage in what is important or meaningful to oneself. Indeed, increased engagement in what is important can precede reductions in a person’s suffering [75]. While digging deeper into what makes a meaningful social interaction low or high quality is a question reserved for future research and clinical work, we propose that learning more about what reinforces engagement in meaningful social interactions might contribute to people having more high-quality social interactions.

## Supporting information

S1 Table Response by group to the item “How many social interactions were meaningful to you?” within one 3-hour time window in relative (%) and absolute (n) numbers. (DOCX)

S2 Table Response by group to the items “Did you perceive the interaction as pleasant?,” on a scale of 0–100 (unpleasant to pleasant), and “How would you estimate the level of intimacy of the interaction?” on a scale of 0–100 (not intimate to intimate) within one 3-hour time window in relative (%) and absolute (n) numbers. Pleasantness and intimacy of meaningful social interactions were combined into an estimation of the quality of social interactions.
(DOCX)

S3 Table Response by group to the item “Did you wish for such a [meaningful] social interaction?” (No, Yes, I don’t know) within one 3-hour time window in relative (%) and absolute (n) numbers. (DOCX)

S4 Table Response by group to the item “Did you avoid such a [meaningful] social interaction?” (No, Yes, I don’t know) within one 3-hour time window in relative (%) and absolute (n) numbers. (DOCX)

S1 Data (CSV)

S2 Data (R)
