# Supplementary material for: Having versus not having social interactions in patients diagnosed with depression or social phobia and controls
Source: PLoS One. 2021 Apr 14;16(4):e0249765. doi: 10.1371/journal.pone.0249765 (PMC8046242; doi:10.1371/journal.pone.0249765)
Supplement: S1 Table — (DOCX) [file pone.0249765.s001.docx]

**S1 Table. Response by group to the item “How many social interactions were meaningful to you?” within one 3-hour time window in relative (%) and absolute (n) numbers.**

|  | 0 | | 1 | | 2 | | 3 | | 4 | | 5 | | 5+ | | SUM | |
| --- | --- | --- | --- | --- | --- | --- | --- | --- | --- | --- | --- | --- | --- | --- | --- | --- |
| **Groups** | % | *n* | % | *n* | % | *n* | % | *n* | % | *n* | % | *n* | % | *n* | % | *n* |
| **MDD** | 19.84 | 550 | 50.65 | 1404 | 19.16 | 531 | 5.95 | 165 | 1.26 | 35 | 0.97 | 27 | 2.16 | 60 | 100.00 | 2772 |
| **SP** | 20.43 | 221 | 50.46 | 546 | 19.78 | 214 | 5.36 | 58 | 2.03 | 22 | 0.37 | 4 | 1.57 | 17 | 100.00 | 1082 |
| **CG** | 18.80 | 585 | 46.77 | 1455 | 20.93 | 651 | 6.46 | 201 | 3.02 | 94 | 1.35 | 42 | 2.67 | 83 | 100.00 | 3111 |

MDD = Major Depressive Disorder, SP = Social Phobia, CG = Control Group.
