# Supplementary material for: Having versus not having social interactions in patients diagnosed with depression or social phobia and controls
Source: PLoS One. 2021 Apr 14;16(4):e0249765. doi: 10.1371/journal.pone.0249765 (PMC8046242; doi:10.1371/journal.pone.0249765)
Supplement: S2 Table — Pleasantness and intimacy of meaningful social interactions were combined into an estimation of the quality of social interactions. (DOCX) [file pone.0249765.s002.docx]

**S2 Table. Response by group to the items “Did you perceive the interaction as pleasant?,” on a scale of 0–100 (unpleasant to pleasant), and “How would you estimate the level of intimacy of the interaction?” on a scale of 0–100 (not intimate to intimate) within one 3-hour time window in relative (%) and absolute (n) numbers. Pleasantness and intimacy of meaningful social interactions were combined into an estimation of the quality of social interactions.**

|  | MDD | | SP | | CG | |
| --- | --- | --- | --- | --- | --- | --- |
|  | Mean | SD | Mean | SD | Mean | SD |
| Pleasantness | 73.29 | 24.30 | 68.43 | 23.20 | 84.82 | 18.10 |
| Intimacy | 76.80 | 23.50 | 70.80 | 23.50 | 81.50 | 20.40 |

MDD = Major Depressive Disorder, SP = Social Phobia, CG = Control Group.
