# Supplementary material for: Having versus not having social interactions in patients diagnosed with depression or social phobia and controls
Source: PLoS One. 2021 Apr 14;16(4):e0249765. doi: 10.1371/journal.pone.0249765 (PMC8046242; doi:10.1371/journal.pone.0249765)
Supplement: S3 Table — (DOCX) [file pone.0249765.s003.docx]

**S3 Table. Response by group to the item “Did you wish for such a [meaningful] social interaction?” (No, Yes, I don’t know) within one 3-hour time window in relative (%) and absolute (n) numbers.**

|  | Yes | | No | | I don’t know | | SUM | |
| --- | --- | --- | --- | --- | --- | --- | --- | --- |
| **Groups** | % | *n* | % | *n* | *%* | *n* | % | *n* |
| **MDD** | 12.01 | 66 | 65.26 | 359 | 22.73 | 125 | 100.00 | 550 |
| **SP** | 19.89 | 44 | 49.36 | 109 | 30.76 | 68 | 100.00 | 221 |
| **CG** | 12.47 | 73 | 63.07 | 369 | 24.46 | 143 | 100.00 | 585 |

MDD = Major Depressive Disorder, SP = Social Phobia, CG = Control Group.
