# Supplementary material for: Having versus not having social interactions in patients diagnosed with depression or social phobia and controls
Source: PLoS One. 2021 Apr 14;16(4):e0249765. doi: 10.1371/journal.pone.0249765 (PMC8046242; doi:10.1371/journal.pone.0249765)
Supplement: S4 Table — (DOCX) [file pone.0249765.s004.docx]

**S4 Table. Response by group to the item “Did you avoid such a [meaningful] social interaction?” (No, Yes, I don’t know) within one 3-hour time window in relative (%) and absolute (n) numbers.**

|  | Yes | | No | | I don’t know | | SUM | |
| --- | --- | --- | --- | --- | --- | --- | --- | --- |
| **Groups** | % | *n* | % | *n* | *%* | *n* | % | *n* |
| **MDD** | 21.45 | 118 | 64.92 | 357 | 13.63 | 75 | 100.00 | 550 |
| **SP** | 22.15 | 49 | 64.72 | 143 | 13.13 | 29 | 100.00 | 221 |
| **CG** | 13.51 | 79 | 79.49 | 465 | 7.00 | 41 | 100.00 | 585 |

MDD = Major Depressive Disorder, SP = Social Phobia, CG = Control Group.
